# Multimodal and autoregulation monitoring in the neurointensive care unit

**DOI:** 10.3389/fneur.2023.1155986

**Published:** 2023-04-20

**Authors:** Jeffrey R. Vitt, Nicholas E. Loper, Shraddha Mainali

**Affiliations:** ^1^Department of Neurological Surgery, UC Davis Medical Center, Sacramento, CA, United States; ^2^Department of Neurology, UC Davis Medical Center, Sacramento, CA, United States; ^3^Department of Neurology, Virginia Commonwealth University, Richmond, VA, United States

**Keywords:** neuromonitoring, transcranial Doppler, multimodal monitoring, near infrared spectroscopy, PbtO_2_ = brain tissue oxygen tension, cerebral autoregulation, neurovascular coupling, pressure reactivity index

## Abstract

Given the complexity of cerebral pathology in patients with acute brain injury, various neuromonitoring strategies have been developed to better appreciate physiologic relationships and potentially harmful derangements. There is ample evidence that bundling several neuromonitoring devices, termed “multimodal monitoring,” is more beneficial compared to monitoring individual parameters as each may capture different and complementary aspects of cerebral physiology to provide a comprehensive picture that can help guide management. Furthermore, each modality has specific strengths and limitations that depend largely on spatiotemporal characteristics and complexity of the signal acquired. In this review we focus on the common clinical neuromonitoring techniques including intracranial pressure, brain tissue oxygenation, transcranial doppler and near-infrared spectroscopy with a focus on how each modality can also provide useful information about cerebral autoregulation capacity. Finally, we discuss the current evidence in using these modalities to support clinical decision making as well as potential insights into the future of advanced cerebral homeostatic assessments including neurovascular coupling.

## Introduction

In patients with acute brain injury (ABI), there is a diverse and heterogenous range of pathologic processes that can often lead to irreversible neurologic insult. Following the primary injury, a cascade of maladaptive and deleterious physiologic processes can ensue in the form of edema, seizures, spreading cortical depolarization, metabolic failure, neuro-inflammation as well as impaired cerebrovascular reactivity, leading to cerebral injury and subsequent cellular death (1, 2). Prevention of secondary brain injury therefore is of paramount importance in neurocritical care with the goal of optimizing conditions to maximize the potential for recovery. Traditionally, management decisions have been guided by neurologic examination and neuroimaging; and while both provide invaluable clinical insights, in isolation, these approaches do not provide an understanding of the ongoing dynamic pathobiological processes, which when monitored can guide medical intervention in real time. As such, there has been an increased focus over the past few decades on the development and utilization of neuromonitoring techniques that allow for enhanced surveillance of cerebral physiologic parameters in order to detect early signs of secondary brain injury and allow for goal directed interventions to stave off irreversible damage (3).

Though advances in neuromonitoring represent a major breakthrough in the field of neurocritical care and provide new insights in the complexity of ABI pathophysiology, a single neuromonitoring device is insufficient in providing a comprehensive scope of the intricate and dynamic nature of impaired cerebral physiology (4, 5). Subsequently focus has shifted to bundling complementary neuromonitoring techniques, termed multimodality monitoring (MMM), to enhance the predictive value of the physiologic outputs and allow for individualized patient management decisions (4, 6). One of the advantages of the expanded use of MMM is the ability to characterize cerebral autoregulation (CA) capacity, which has been validated as an important prognostic indicator and may be useful to guide hemodynamic decisions, with the goal of optimizing cerebral perfusion (7, 8).

**Figure 1:**
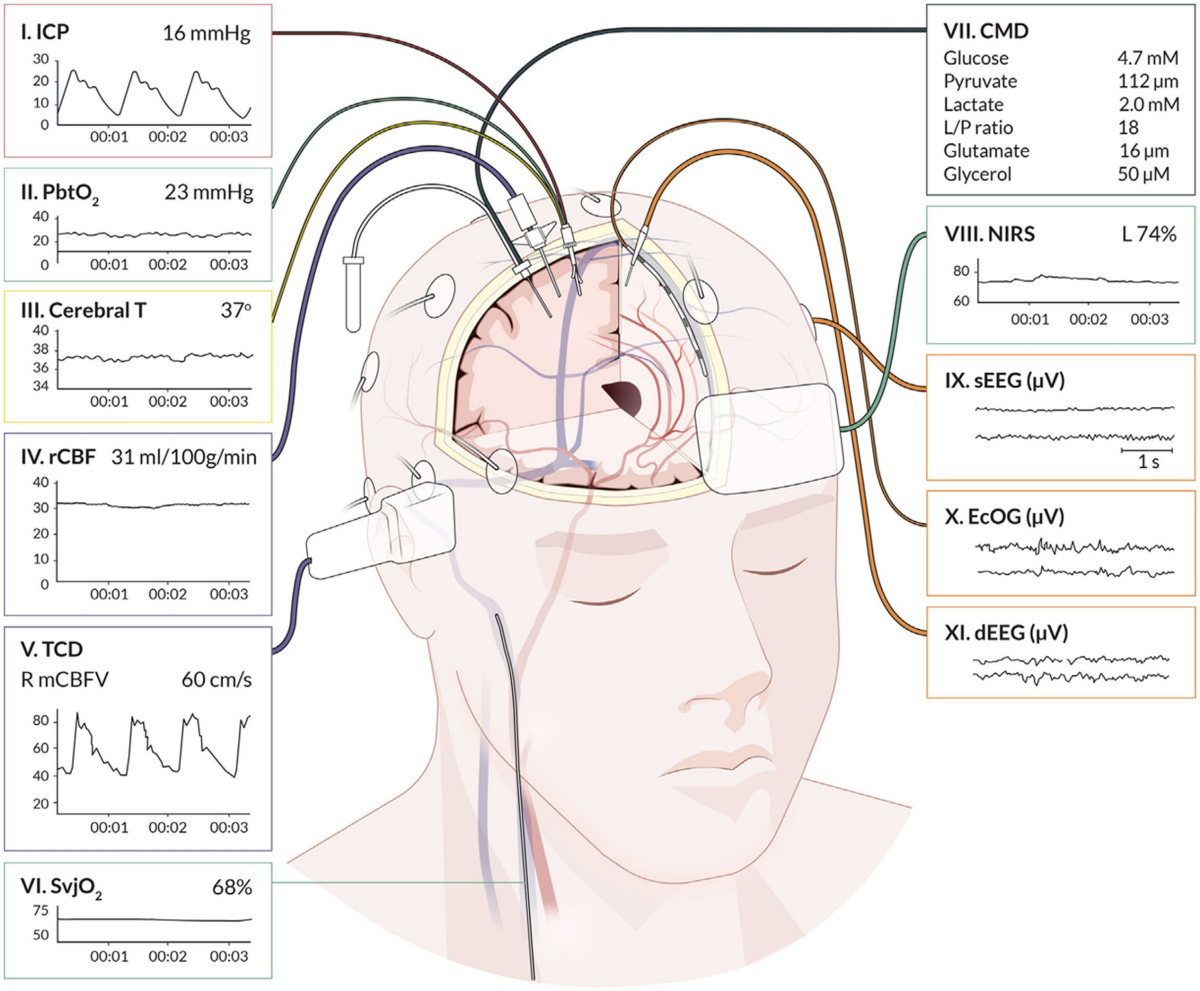
Graphical representation of cerebral multimodality monitoring modalities. Cerebral T, cerebral temperature; CMD, cerebral microdialysis; dEEG, depth electroencephalography; ECoG, electrocorticography; ICP, intracranial pressure; NIRS, near-infrared spectroscopy; PbtO_2_, partial pressure of brain tissue oxygenation; rCBF, regional cerebral blood flow; sEEG, surface electroencephalography; SvjO_2_, jugular bulb venous oximetry; TCD, transcranial Doppler. Reprinted with permission from open access publication corresponding author, Tas and colleagues (4). Professional illustration by Anna Sieben (Sieben Medical Art).

This multifaceted approach has begun to reshape the landscape of neurocritical care by moving away from standardized “one size fits all” treatment strategies to individualized precision medicine; however, questions still exist including how to best integrate and interpret the high dimensionality of signals and whether such an approach will result in improved patient outcomes (3, 9). Furthermore, when interpreting monitoring data, it is important to recognize potential limitations of each modality and specific characteristics including whether it provides continuous or intermittent assessment as well as if the device is measuring focal or global cerebral parameters (10, 11). Common neuromonitoring tools employed in the neurologic intensive care unit (ICU) are highlighted in Figure 1. This review will provide the most recent update on MMM with a focus on invasive and non-invasive monitoring strategies that can be used to simultaneously provide CA assessment. Modalities that do not possess capabilities for CA evaluation, such as quantitative electroencephalography (qEEG) and cerebral microdialysis, will not be covered.

## Cerebral physiology

Advances in neuromonitoring capabilities has allowed for enhanced understanding of complex cerebral physiologic relationships, both in normal homeostasis and with harmful derangements, with the potential to drive clinical management decisions. An understanding of major cerebral physiologic concepts is crucial for proper interpretation of MMM outputs and informed decision making for cerebral optimization.

### Pressure-volume relationship

As the brain resides within the rigid and inflexible cranial vault, alterations in total volume result in corresponding changes in intracranial pressure (ICP). As described in the Monroe-Kellie doctrine, an increase in any of the components of the intracranial system, namely brain, blood and cerebrospinal fluid (CSF), must be accompanied by an equivalent volumetric reduction in another constituent to maintain homeostasis (12). Disproportionate increases in volume (such as cases of hematoma formation or acute hydrocephalus) are rapidly met with exhausted compliance and the pressure-volume relationship becomes non-linear where small alterations in volume induce large changes in ICP (12–14).

Examination of the ICP waveform can provide valuable insights into intracranial compliance and risk for deterioration (15, 16). In the absence of significant pathology, the ICP waveform has three notches corresponding to the systolic (P1), tidal (P2), and dicrotic waves (P3) and are of decreasing amplitude (16). As intracranial compliance decreases, P2 and P3 begin to exceed P1 and eventually P3 disappears leaving a sinusoidal morphology (12, 16). Elevation of P2 can predict risk for subsequent ICP crisis and during periods of severely elevated ICP there is often a reduction in ICP waveform complexity (15, 17).

### Vasomotor reactivity

Under normal conditions, the brain requires a constant cerebral blood flow (CBF) of ~50–60 mL/100 g/min to maintain normal metabolic and physiologic conditions (18). Regulation of CBF involves a complex interplay between various cellular signaling pathways that act to modulate vascular tone to ensure optimal perfusion and homeostasis. The cerebrovasculature is particularly sensitive to changes in arterial blood carbon dioxide (PaCO_2_), pH, and to a lesser extent oxygen, through a process termed vasomotor reactivity (VMR) (19). PaCO_2_ acts as a fundamental regulator of CBF, with an ~3% increase in CBF for every 1 mmHg increase in PaCO_2_, through modulation of arteriolar tone by nitric oxide production and alterations in intracellular smooth muscle hydrogen and calcium ion concentrations (20, 21). Avoiding hypercarbia is particularly important for patients with reduced intracranial compliance and elevated ICP due to the potential for increase total cerebral blood volume (CBV) resulting from vasodilation (22). Excessive hyperventilation on the other hand, can dramatically reduce ICP through hypocapnic induced vasoconstriction, however may result in cerebral ischemia and secondary brain injury from neuronal ecotoxicity and diminished CBF (23). While not as potent a modulator of vascular tone under normal physiologic conditions as PaCO_2_, CBF is also impacted by changes in arterial blood oxygen (PaO_2_) with vasodilation occurring when arterial tension falls below 50 mmHg to protect against cerebral hypoxia (24).

### Cerebral autoregulation

**Figure 2:**
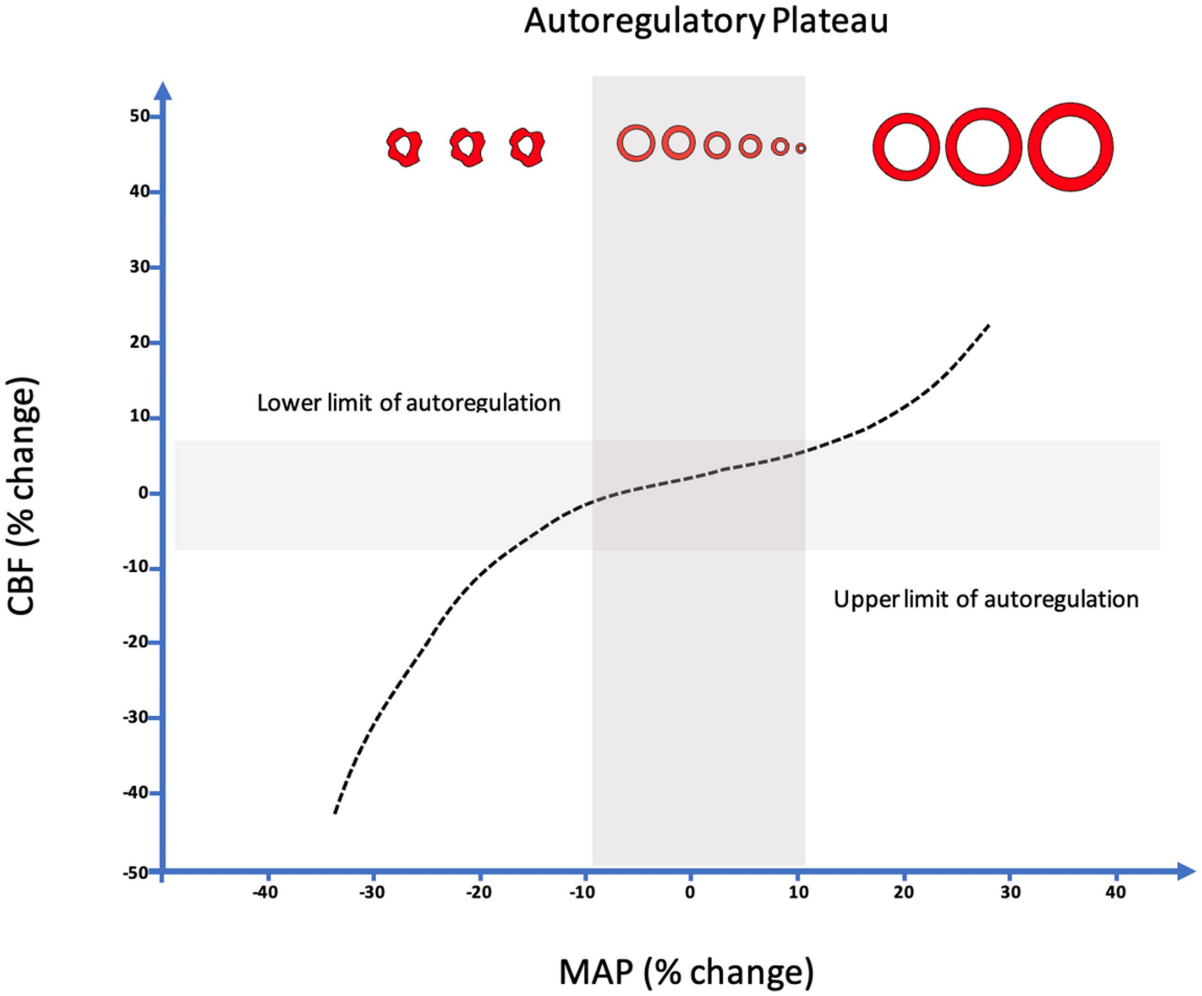
Relationship between changes in MAP from baseline (Δ%MAP) and concomitant relative changes in CBF (Δ%CBF). Upper and lower limits of autoregulation demonstrated by linear correlation between MAP and CBF indicative of pressure passive state. NB. The classic Lassen’s curve demonstrating a wide range of autoregulation (~50–150 mm hg) has been challenged. It is suggested that a narrow range of autoregulation (~15–200 mm hg) is likely to be more common, even in healthy individuals, and is influenced by the rate of arterial pressure changes.

Autoregulatory status has gained recognition as a crucial protective homeostatic mechanism and an important determinant of mortality and functional outcome in patients with diffuse brain injury such as severe traumatic brain injury (TBI) and subarachnoid hemorrhage (SAH) (8, 25–28). First described in humans by Lassen and colleagues in 1959, CA describes the capacity of the cerebral pre-capillary arterioles to relax and constrict to changes in transmural pressure in order to ensure a constant cerebral blood flow (CBF) over a range of mean arterial pressures (MAP) (29). More recent investigations have demonstrated that the range of MAP during which CA remains intact is narrower than previously thought, even in otherwise healthy individuals, and is influenced by the rate of arterial pressure changes (Figure 2) (30). In the setting of ABI, the CA plateau may be further restricted, shifted or all together exhausted thereby predisposing patients to periods of ischemia or conversely hyperemia whenever MAP falls outside the lower and upper limits of autoregulation, respectively (31). In such patients cerebral ischemia may occur under ranges of MAP typically considered normal, thus highlighting the potential significance of individualized assessment and optimization (26, 31, 32).

Early studies evaluating CA capacity in ABI focused on intermittent methods for CA determination including various neuroimaging modalities such as positron emission tomography (PET) and computed tomographic xenon (XE-CT) as well as indirect CBF estimation with transcranial doppler (TCD) in response to alterations in MAP either with vasopressors or non-invasively with techniques such as thigh-cuff deflation and orthostatic hypotension provocation (10, 33). More recently, novel methods have been developed for continuous assessment of CA indices based on CBF responses to spontaneous changes in MAP or cerebral perfusion pressure CPP and are outlined in Table 1 (44). These advances have enhanced the clinical applicability of CA and provided a foundation to develop monitoring and treatment protocols based off CA capacity (45). While there is of yet no primary data from large scale randomized controlled trials to support the use of CA monitoring, growing evidence from retrospective and prospective analysis suggests that it may provide crucial insights into the complexities of deranged cerebral physiology inherent in ABI patients and serve as a vital tool to drive clinical management decisions (26, 46–48). This is the topic of several ongoing clinical investigations that will hopefully provide further evidence of how continuous CA assessment may be used to drive patient care decisions (NCT03987139, NCT05670028, NCT02351518).

**Table 1:** Neuromonitoring Devices, Physiologic Outputs and Autoregulation Indices.

Modality	Description	Primary signal output	Relevant thresholds	Cerebral autoregulation indices	Signals correlated	Thresholds
Intracranial pressure monitoring	Invasive insertion into cranium. Most commonly in the ventricular or parenchymal space.	ICP CPP	≤20 or 22 mmHg (34) 60–70 mmHg (34)	PRx	ICP and MAP	>0.2 Mortality (13) >0.05 Unfavorable Outcome (13)
Brain tissue oxygenation	Invasive probe measuring oxygen tension in brain parenchyma. Reflects the balance between cerebral oxygen delivery, diffusion and demand.	PbtO_2_	>15–20 mmHg (35)	ORx	PbtO_2_ and CPP	>0.3–0.4 Unfavorable Outcome (14)
Transcranial doppler	Non-invasive low frequency ultrasound capable of detecting proximal cerebral vessel blood flow	CBFV PSV EDV MFV PI	MCA MFV >160–200 cm/s suggests vasospasm in SAH (36, 37) MFV < 40 cm/s or EDV <20–25 cm/s associated with worse outcome in TBI (38, 39) PI>1.3–1.5 associated with worse outcome in TBI (40, 41)	Sx Sx_a Mx Mx_a	PSV and CPP PSV and MAP MFV and CPP MFV and MAP	>−0.2 Mortality (25) >−0.15 Unfavorable Outcome >0.05 Mortality (25) >−0.1 Unfavorable Outcome >0.3 Mortality (25) >0.3 Unfavorable Outcome >0.3 Unfavorable Outcome (25)
Near infrared spectroscopy	Non-invasive near infrared light source sensitive to oxygenation status of hemoglobin with ability to describe cerebral oxygenation and blood flow	rSO_2_ TOI	Absolute values <50–60% (42) or decline in baseline by >13% concerning for ischemia (43)	COx TOI THx	rSO_2_ and MAP TOI and MAP HbT and MAP	Limited clinical evidence to support clear thresholds In general (+) values have been considered consistent with impaired autoregulation

ICP, Intracranial pressure; CPP, cerebral perfusion pressure; PRx, pressure reactivity index; MAP, mean arterial pressure; PbtO_2_, Brain tissue partial pressure oxygenation; ORx, Brain tissue oxygen reactivity index; CBFV, Cerebral blood flow velocity; PSV, peak systolic velocity; EDV, end diastolic velocity; MFV, mean flow velocity; PI, Pulsatility Index; SAH, subarachnoid hemorrhage; TBI, traumatic brain injury; Sx/Sx_a, systolic flow index; Mx/Mx_a, mean flow index; rSO_2_, regional cerebral oxygen saturation; TOI, tissue oxygenation index; COx, cerebral oximetry index; TOI, tissue oxygen reactivity index; THx, total hemoglobin index; HbT, total hemoglobin concentration.

### Neurovascular coupling

While there is mounting evidence regarding the integral role of CA, both as a prognostic marker and foundation for individually targeted management, the brain possesses other homeostatic mechanisms of equal or potentially greater importance for cerebral reserve and guarding against irreversible injury (49). The brain is a highly metabolic organ, utilizing more than 20% of bodily oxygen and glucose despite comprising only 2% of body weight, and has limited capacity for intracellular energy storage thus rendering it dependent on a continuous and tightly regulated blood supply to regions with higher metabolic demand (50, 51). The governing mechanism regulating CBF in response to neuronal activity is termed neurovascular coupling (NVC) and is crucial for ensuring precise cerebral metabolic demands are met (52). NVC has been studied in a variety of conditions with evidence of impairment in chronic conditions such as hypertension, atrial fibrillation and Alzheimer’s Disease leading to blunted NVC response and oxidative stress (49, 53). Impaired NVC may also play a role in the development of delayed cerebral ischemia (DCI) with SAH animal models demonstrating impaired NVC throughout the acute period following aneurysm rupture, leading to an imbalance between metabolic demand and CBF, and rendering the brain vulnerable to ischemia in the face of spreading cortical depressions (54). In acute ischemic stroke (AIS) animal models, early impairment of NVC is associated with reduction in neuronally mediated feed-forward regulation of CBF as well as evidence of prolonged NVC disruption involving territories outside the zone of ischemia that does not resolve with the restoration of CBF, thus contributing to ongoing cerebral dysfunction and injury accumulation (52). Similar findings have been reported in human subjects, where global NVC dysfunction is described following AIS and correlated with the degree of vessel stenosis in addition to functional outcomes (34, 55, 56).

The majority of NVC research has focused on outpatient methods of assessment that rely on patient participation to perform cognitive, verbal or motor tasks with evaluation of a CBF response measured by non-invasive modalities such as TCD and advanced perfusion imaging (50, 52). While these methods are generally not suitable for patients in the ICU environment, several physiologic approaches leveraging MMM have been developed and allow for NVC assessment and recognition of disturbed cerebral physiology in critically ill patients. These methods often involve fast Fourier transformation of EEG signal into frequency bands to represent neuronal activity combined with a method of CBF estimation including ICP pulse-waveform, near infrared spectroscopy (NIRS) and TCD alterations in response to electrocortical activity (57–61). Though these approaches have largely been investigated in small cohorts as proof of principle studies, a recent investigation involving nine comatose patients with various forms of ABI were studied using NIRS-EEG and found normalization of NVC predicted the recovery of consciousness with >99% accuracy (62). Though these results need to be validated in a larger and more diverse cohort, they highlight the promising role NVC may play in improving our understanding of disturbed cerebral physiology across a spectrum of neurologic disorders including disorders of consciousness. It remains to be seen whether enhanced processes for monitoring and identifying disturbed NVC will provide a foundation to develop timely targeted interventions to ameliorate pathologic processes or refine our approach to prognostication.

## Intracranial pressure monitoring

ICP monitoring was first described by Lundberg and colleagues in 1964 and has since become the most widely employed invasive monitoring technique with the largest amount of supporting data (4, 63). Elevations in ICP are directly related to excess mortality and poor neurologic outcomes, likely through destructive mechanical forces as well as reduced cerebral perfusion leading to ischemia and oligemia within vulnerable brain parenchyma (32, 64–66). The two most utilized invasive modalities for ICP monitoring are intraventricular catheters and parenchymal monitors, both of which can provide continuous ICP data whereas the former also allows for therapeutic CSF diversion. In general, these modalities are considered monitors of global ICP given pressure equilibration in the intracranial vault, however, in certain conditions such a unilateral masses with associated large midline shift or posterior fossa or infratentorial lesions, there may be an associated pressure gradient leading to differential ICP measurements depending on catheter tip placement (67).

ICP monitoring is a central focus in the management of severe TBI and recommended (Level IIB) by the Brain Trauma Foundation (BTF) for all patients with a Glasgow Coma Scale (GCS) 3–8 and abnormal CT scan as well as for patients who have a normal CT head but meet at least two of the three following criteria: age >40 years, unilateral or bilateral motor posturing, or systolic blood pressure (SBP) <90 mmHg (68). Furthermore, the International Multidisciplinary Consensus Conference on MMM in Neurocritical Care recommends ICP monitoring to better contextualize and interpret data generated from other monitoring devices given the robust level of evidence supporting its use (3). Adherence to a protocolized ICP management strategy in severe TBI has been demonstrated to decrease 2-week adjusted mortality and the recently published multinational SYNAPSE-ICU study found that ICP monitoring and management in a mixed cohort of ABI patients was associated with increased therapeutic intensity as well as improved neurologic outcome and 6-month mortality, with the largest magnitude of benefit in patients with more severe grade injury (69–71).

**Figure 3:**
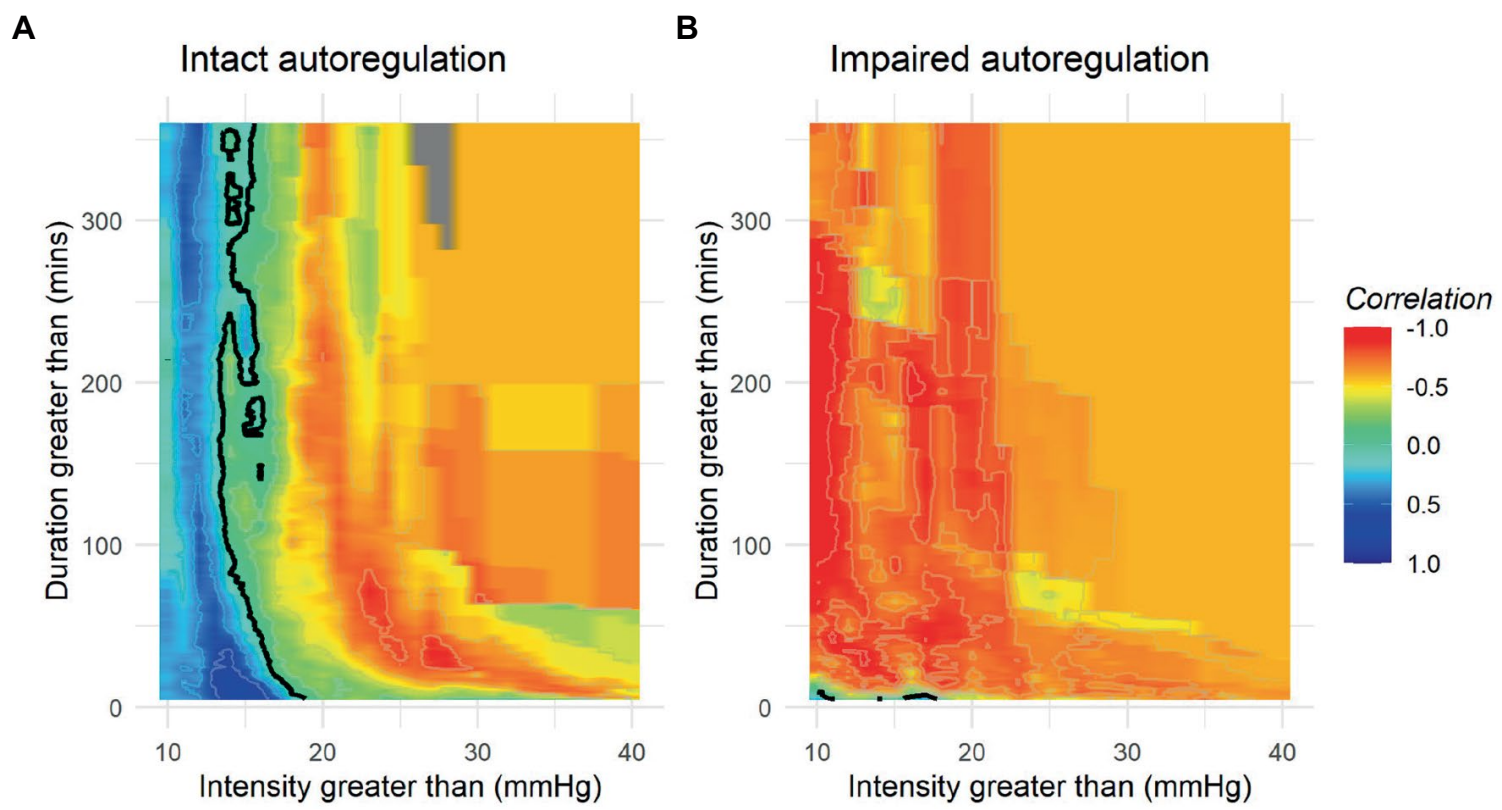
Population based incracranial pressure (ICP) intensity map stratified by autoregulatory status. Red areas indicate areas where ICP intensity is associated with poor outcome, while blue areas indicate good outcomes. Patients with intact autoregulation tolerate longer durations and intensities of ICP elevation compared to those with impaired autoregulation where no safe zone could be identified. **(A)** Intact autoregulation (mean PRx <= 0.3), **(B)** Impaired autoregulation (mean PRx > 0.3). Reprinted with permission from open access publication corresponding author, Akerlund and associates (52).

While it seems evident that avoidance of elevated ICP would be beneficial in ABI patients, efforts to define a precise treatment threshold for ICP have yielded variable results (66, 72, 73). In the 2000 BTF guidelines, an ICP threshold of 20–25 mmHg was recommended and later refined to less than or equal to 20 mmHg in 2007 (74). In most recent 2016 published guidelines, further adjusted the recommended threshold to <22 mmHg, largely based on a single retrospective analysis of 459 severe TBI patients where 22 mmHg best predicted the intersection between mortality and favorable outcome (26, 68). Subgroup analysis in this study found a threshold of 18 mmHg was more accurate for female and elderly patients, highlighting the heterogeneity present in subpopulations, and further studies have demonstrated that ICP values as low as 10 mmHg may still confer harm in some patients raising the question if a single “one size fits all” threshold is suitable (26, 75). These questions are further magnified by the results of the BEST TRIP trial where a management strategy solely focused on maintaining ICP at 20 mmHg or less was not shown to be superior to care based on imaging and clinical examination (76). More recently, the concept of a pressure-dose model has arisen which weighs both the magnitude and duration of pathological ICP to help inform treatment decisions (Figure 3) (32, 64, 77). According to this model, even ICP values below guideline recommended treatment thresholds (15–20 mmHg) can induce injury if sufficiently sustained whereas in one study, ICP values above 20 mmHg and 30 mmHg correlate with worse outcome after 37 and 8 min, respectively (32). These findings suggest that ICP should not be viewed as a dichotomous parameter with values below a particular threshold regarded as safe and those above considered dangerous in all patient populations. Moreover, this research has illuminated how vulnerability to ICP-related injury largely depends on CA status where even small elevations are poorly tolerated when CA is compromised (32, 78).

### Cerebral perfusion pressure

Another important physiologic parameter indirectly measured from ICP monitoring is CPP, expressed by MAP-ICP, and reflects the driving pressure to the brain parenchyma. The BTF recommends maintaining CPP between 60 and 70 mmHg based on historical observational data demonstrating worse outcomes with lower thresholds as well as evidence of heightened risk for acute respiratory distress syndrome (ARDS), likely due to increased vasopressor use and fluid balance, when uniformly targeting CPP above 70 mmHg (8, 79, 80). Modest elevations in CPP have been shown to have a protective effect against ICP insults. However, recent investigations have demonstrated the ideal range of individualized CPP appears to be quite variable and highly dependent on CA status with evidence of average CPP <70 mmHg being poorly tolerated in those with impaired CA (26, 31, 32).

### Pressure reactivity index

**Figure 4:**
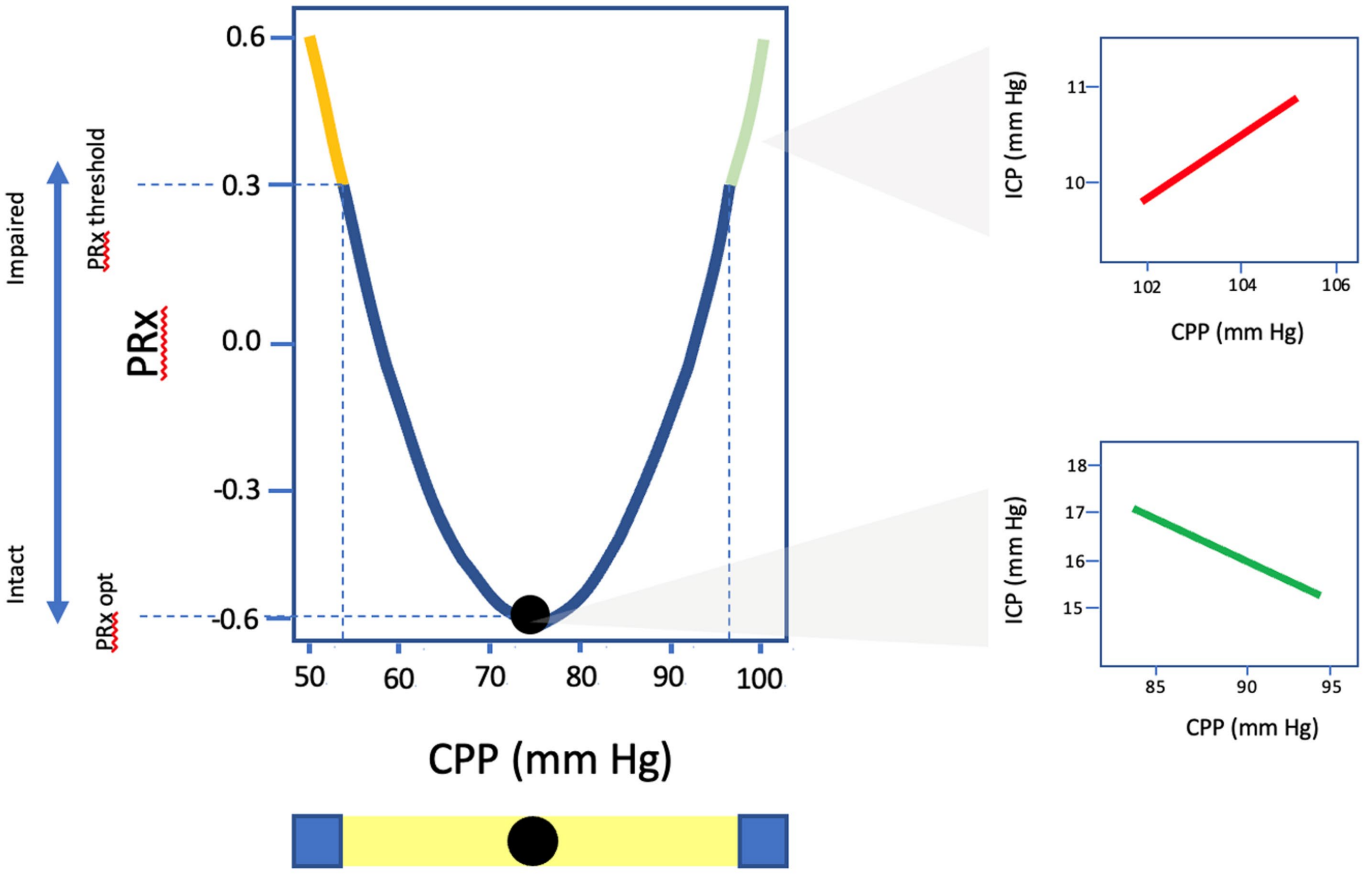
Graphic representation of U-shaped curve of PRx values as a function of CPP. CPP_opt_ identified as the CPP value with the lowest associated PRx and most optimal autoregulatory state. PRx, pressure reactivity; CPP, cerebral perfusion pressure; ICP, intracranial pressure.

CA capacity can be assessed by the Pressure Reactivity Index (PRx), which is a moving Pearson’s correlation that expresses the relationship between slow-wave changes in ICP, a surrogate marker of pulsatile CBV, in response to alterations in MAP/CPP where positive values denote impaired CA and negative values suggest intact cerebrovascular reactivity (13). Multiple studies have validated the prognostic importance of PRx in TBI populations with thresholds of +0.05 and +0.25 for poor neurologic outcome and mortality, respectively (7, 26, 81). Certain TBI populations appear particularly susceptible to impaired CA following TBI as measured by PRx including the elderly as well as those with diffuse injury patterns (47, 82). Additionally, elevated PRx has been correlated with impaired cerebral metabolism and energy utilization in addition to expansion of cerebral edema following parenchymal contusion (83, 84). Trending continuous PRx as a function of ICP fluctuations recently has been used to assign individual ICP thresholds, where ICP values at which PRx is >+0.2, was found to have superior predictive value compared to static thresholds for mortality and functional outcomes (85, 86). Using a similar methodology, plotting PRx against CPP allows for the determination of optimal CPP (CPP_OPT_), where CPP values with the lowest associated PRx are interpreted as most ideal for preservation of autoregulatory status and a target for hemodynamic management (Figure 4) (7, 8). TBI patients with a median CPP that more closely approximates CPP_OPT_ have been found to have improved cerebral oxygenation, more favorable markers of cerebral energy metabolism, and better clinical outcomes (25, 87–90). Recently, the Phase II COGITATE study published their results and highlighted the safety and feasibility of a CPP_OPT_ guided protocol compared to BTF guideline recommendations of CPP 60–70 mmHg (46). Though not powered for outcomes, fewer patients died in the intervention arm (23% vs. 44%) with no increased in therapeutic intensity level or adverse events suggesting that such a strategy is achievable and safe in clinical practice and may improve functional recovery in TBI by ensuring adherence to individualized optimal physiologic targets.

Though PRx and CPP_OPT_ targeted therapy hold promising potential to transform the landscape of neurocritical care management and are the most well-established of all the continuous CA indices, there are several important limitations and questions that must be acknowledged. PRx requires exhausted intracranial compliance in order to visualize changes in ICP in response to minimal alterations in CBV and therefore may provide inconclusive results in patients lacking significant mass effect or with highly compliant cranial compartments such as seen in age-associated atrophy or following decompressive hemicraniectomy (35, 47, 91). Just as ICP monitoring represents a global evaluation of pressure, PRx represents a global estimator of CA and therefor is unable to characterize heterogeneous patterns of disturbed CA (90, 92). Furthermore, PRx requires high-frequency signal processing, on the level of second-by-second data acquisition which is labor intensive, expensive, and not widely available for clinical monitoring outside of several specialized academic centers. As a result, CPP_OPT_ can be challenging to estimate in a significant number of individuals, particularly in the elderly, with 16–45% of monitored 4–6 h epochs in retrospective analysis incapable of generating a CPP_OPT_ (25, 35, 89, 91). To address this issue, several studies have explored the use of minute-by-minute low-resolution PRx compared to standard PRx, finding that it is comparable in regards to outcome prediction and CPP_OPT_ determination albeit with slightly lower precision (81, 93). These advances have the potential to expand the adoption of these monitoring approaches across a broader range of clinical settings lacking high-resolution signal monitoring however, widespread implementation cannot be expected until clinical efficacy is proven (90). Furthermore, innovations in non-invasive approaches to neuromonitoring such as automated robotic TCD has the potential to provide analogous and complementary information in ABI populations (94).

## Cerebral oxygen monitoring

Though ICP/CPP monitoring plays a crucial role in the management of ABI, it does not provide information about the adequacy of cerebral perfusion and the underlying metabolic demand (CMRO_2_). Following ABI, derangements in CBF and oxidative metabolism are common and contribute to secondary brain injury in the form of cerebral hypoxia and metabolic crisis, independent of ICP and CPP (95–97). PET studies in patients with TBI reveals evidence of regional microvascular collapse leading to decreased diffusion capacity and ischemia occurring both in areas with damage and those that appear structurally normal on imaging (98). In clinical studies, the magnitude and duration of cerebral hypoxia has been shown to correlate with worse neurologic outcomes and increased mortality making it an attractive target for neuromonitoring (97, 99). The two most widely used modalities for cerebral oxygenation include jugular venous oxygen saturation (SjvO_2_), which measures the balance between global CBF and CMRO_2_, and brain tissue oxygen partial pressure (PbtO_2_) that provides information regarding CBF in addition to oxygen diffusion and delivery (100). As SjvO_2_ monitoring is not capable of continuous CA assessment and has largely fallen out of favor in most centers, this section will focus exclusively on PbtO_2_ (101).

### Brain tissue oxygen partial pressure

Monitoring PbtO_2_ requires surgical placement, often into the subcortical white matter, and detects focal changes in oxygen tension (102, 103). PbtO_2_ is a complex and multidimensional physiologic parameter that reflects CBF in addition to oxygen delivery, diffusion and consumption (100). Normal values are between 23 and 35 mmHg though when <15–20 mmHg and <10 mmHg are considered moderate and severe hypoxia, respectively, with proportionate risk for irreversible cerebral ischemia (103–105). Given the focal nature of PbtO_2_ monitoring, regional variability is common and there is debate on whether monitors should be placed in healthy appearing parenchyma or in perilesional tissue that theoretically is most vulnerable to secondary insults (103, 105). Though values generated from perilesional tissue may provide the best discrimination for neurologic outcome, precise placement of probes is technically challenging and PbtO_2_ response to treatment is often blunted compared to healthy tissue (106, 107). In keeping with this notion, the protocol for the ongoing randomized controlled trial, “Brain Tissue Oxygen Monitoring and Management in Traumatic Brain Injury (BOOST-III)” includes placement of monitors 2 cm from the cortical surface in the least trauma-affected frontal lobe to promote consistency of practice and data interpretation (108). Probe position should always be confirmed on imaging and either FiO_2_ or MAP challenge should be utilized to ensure appropriate function before attempting to interpret data.

Low PbtO_2_ recordings are frequent following ABI, occurring in up to 87% of individuals during monitoring, and influenced by a variety of clinical insults including elevated ICP and suboptimal CPP leading to impaired CBF (65, 95, 109). Multiple studies have also demonstrated the importance of CA in maintaining normal PbtO_2_ with evidence that cerebral hypoxia is more common when CPP falls below PRx derived CPP_OPT_ (88, 110, 111). Ensuring adequate systemic oxygenation is imperative to improving cerebral hypoxia and under usual conditions PbtO_2_ is very responsive to increases in FiO_2_. However, if PbtO2 is not responsive to increase in FiO2, other contributory factors should be assessed such as hemoglobin level, temperature, brain edema and ICP. It is also important to note that prolonged periods of hyperoxia may increase the risk of excitotoxicity (112, 113). In practice, maintaining a minimum PaO_2_ > 90 mmHg or SpO_2_ > 94–98% appears pragmatic to avoid cerebral hypoxia and recently the concept of a brain oxygen (BOx) ratio (PbtO_2_/PaO_2_) was introduced to assist in recognizing altered cerebral physiology even in the setting of normal PbtO_2_ due to overtreatment with FiO_2_ (114, 115). Optimization of PbtO_2_ requires an multidimensional tiered approach involving various measures aimed at correcting hypoxemia, reducing ICP, augmenting CPP and decreasing CMRO_2_ (113, 116).

Treatment response to subthreshold PbtO_2_ is predictive for survival whereas the duration and magnitude of cerebral hypoxia are strongly correlated with increased mortality and poor neurologic outcome (97, 99, 113, 117). In aneurysmal SAH, low PbtO_2_ can predict symptomatic vasospasm though the sensitivity depends heavily on whether the probe location corresponds to the affected vascular territory (118, 119). PbtO_2_ represents the second most commonly used invasive neuromonitor after ICP and the two together are the most widely published MMM combination (4). Bundling PbtO_2_ and ICP/CPP compared to managing ICP/CPP in isolation has been explored in multiple retrospective analyses, both in TBI and SAH populations, and found improved mortality and functional outcomes associated with combined therapy with no increase in length of stay or serious adverse events including ARDS (95, 99, 120–123). Though large prospective trial data is lacking, a 2015 small multicenter study found that PbtO_2_ and ICP/CPP guided therapy was associated with more aggressive ICP control and higher CPP with a trend toward improved functional outcomes at 6 months compared to ICP management alone (124). More recently the BOOST-II trial demonstrated that a protocolized PbtO_2_ and ICP/CPP combined management strategy was safe, feasible and associated with a lower burden of cerebral hypoxia and while not powered for outcome, demonstrated a trend toward lower mortality and better neurologic outcomes (108). Though the data at this time remains too limited to recommend widespread implementation of PbtO_2_ monitoring, based on the promising preliminary studies presented there are several Phase III multicenter randomized controlled trials currently underway, including BOOST-III, BONANZA, and OXY-TC, which will hopefully provide further insights into the role of PbtO_2_ based therapy in severe TBI management (36, 95).

### Brain tissue oxygen pressure reactivity index

Intact CA function appears to exert a protective force in preventing cerebral hypoxia though low PbtO_2_ itself has also been implicated in the development of disturbed cerebrovascular reactivity (37, 110). Several studies have explored the relationship between PbtO_2_ and changes in CPP, termed Brain Tissue Oxygen Pressure Reactivity Index (ORx), which is a moving linear correlation coefficient of PbtO_2_ and CPP data obtained in 10–30 s intervals (37, 125). ORx has been postulated as an index of CA, similar to PRx, with negative values and those near 0 considered intact while disturbed CA is assumed as ORx approaches +1 (27). ORx values were correlated with unfavorable outcomes in a single study with a threshold of +0.3–0.4 and other investigations have demonstrated higher values associated with vasospasm and DCI in SAH patient populations (27, 28). Agreement between ORx and PRx has yielded mixed results with several studies demonstrating concordance between the two indices, particularly when PbtO_2_ is low, though it diverges when PbtO_2_ exceeds 40 mmHg with an increase in ORx despite stable PRx (27, 37). Subsequent investigations have yielded poor correlations between the two indices and an absence of a clear threshold for prognosticating outcomes or ability to determine a CPP_OPT_ based on ORx (125–127). PbtO_2_ is a complex physiologic parameter influenced by a multitude of different systemic and cerebral factors, with CPP being only one aspect, likely hindering precise estimation of CA (116). In a high-resolution TBI dataset, PbtO_2_ failed to demonstrate a reliable response to slow-wave fluctuations in MAP or ICP as would be expected in a surrogate of CA, further calling into question whether PbtO_2_ is an appropriate parameter for CA derivation (125). Furthermore, PbtO_2_ involves monitoring a relatively small focal area of parenchyma, which can exhibit significant regional variability, thereby reducing generalizability and the ability to infer overall CA capacity (105, 106). At present, the data supporting ORx as a reliable surrogate of CA is limited and caution should be exercised before using it clinically. Further investigation is warranted to better understand circumstances when it may be of utility.

## Transcranial Doppler

Transcranial Doppler (TCD) is a non-invasive neuromonitoring technique that has been used in clinical practice since first described by Aaslid and colleagues in 1982 to examine CBF in the basal cerebral arteries (128). CBF velocity (CBFV) and directionality can be determined from the degree of doppler shift created by a moving column of red blood cells through the proximal cerebral vasculature (129). TCD utilizes a low frequency probe (2 MHz) to penetrate the skull with the most common windows for insonation being the transtemporal and suboccipital approaches in which CBFV can be measured in the anterior and posterior circulations, respectively (130). Furthermore, the ophthalmic artery and carotid siphon can be insonated through the transorbital approach and the submandibular window allows for evaluation of the cervical portion of the internal carotid artery (129). While CBFV is proportional to CBF, TCD is unable to accurately account for vessel diameter, making it impossible to draw direct conclusions about CBF as vasodilation and vasoconstriction can also produce reciprocal changes in CBFV (130). Similar to the arterial waveform, the TCD flow velocity (FV) waveform is pulsatile, though lower resistance with continuous flow throughout the cardiac cycle, and allows for quantification of peak systolic velocity (PSV), end diastolic velocity (EDV) and mean flow velocity (MFV) parameters (131).

MFV is very sensitive to corresponding alterations in vessel caliber, making TCD a valuable screening tool for large vessel vasospasm in aneurysmal SAH and TBI (132, 133). Multiple thresholds for grading vasospasm have been proposed with the strongest level of evidence and predictive value found in MFV obtained from the middle cerebral arteries (MCA) (134, 135). MFV exceeding 160–200 cm/s in the MCA has a high positive predictive value (PPV) for moderate to severe angiographic vasospasm while those <120 cm/s are unlikely to have clinically significant spasm (136, 137). The Lindegaard Ratio reflects the MFV of the MCA relative to the ipsilateral ICA and is a useful tool to contextualize elevated velocities more related to vasospasm or hyperemia (138). The rate of vasospasm evolution and overall severity diagnosed on TCD, are established risk factors for the development of DCI, with a 90% sensitivity and 92% negative predictive value (NPV), thus allowing for early targeted intervention to prevent irreversible cerebral infarction and further supports the role of TCD for routine surveillance monitoring in this setting (40, 133, 138). In addition to vasospasm monitoring, TCD is a valuable modality in AIS evaluation with high sensitivity for microembolic signals which can be used for risk stratification of future ischemic events in the setting of carotid stenosis, blunt cerebrovascular injury, as well as cardiac disease and may be useful for guiding decisions for anticoagulation (38, 41, 139, 140).

**Figure 5:**
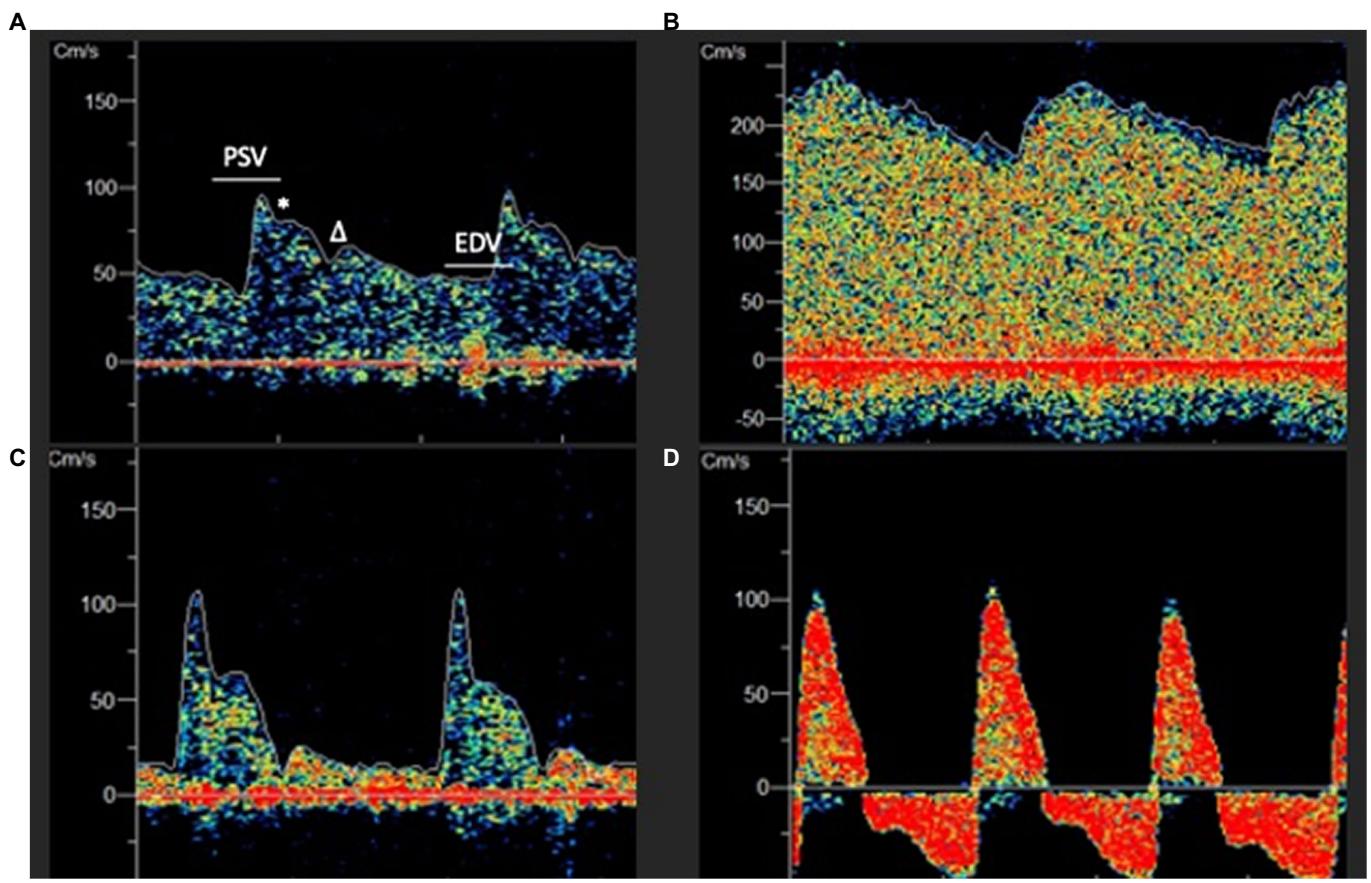
**(A)** Normal appearing low resistance TCD waveform. PSV, Peak systolic velocity; EDV, End Diastolic Velocity; * Windkessel Notch; Δ Dicrotic Notch. **(B)** Hyperemic appearing waveform with elevated EDV, absent dicrotic notch and low pulsatility index (PI). **(C)** High resistance waveform with low EDV and elevated PI. **(D)** Extremely elevated intracranial pressure with peaked systolic upstroke and reversal of diastolic flow in a patient with cerebral circulatory arrest.

Furthermore, FV waveform morphology is sensitive to alterations in CBF and provides valuable insights regarding perfusion status in different vascular beds. For example, elevated ICP characteristically leads to a more pronounced systolic upstroke and loss of the Windkessel notch due to external compression as well as reduced EDV signifying impaired diastolic flow (Figure 5) (131, 141, 142). When ICP exceeds the diastolic closing pressure, a reversal of diastolic CBFV is observed and may eventually progress to cerebrocirculatory arrest characterized by oscillating flow patterns, systolic spikes or absent flow and can be used as an acillary confirmatory test in brain death determination (143). Conversely, in patients with hyperemia due to disturbed CA or arteriovenous malformations, waveforms are typically low-resistance with high EDV relative to PSV and a diminished dicrotic notch (39).

**Figure 6:**
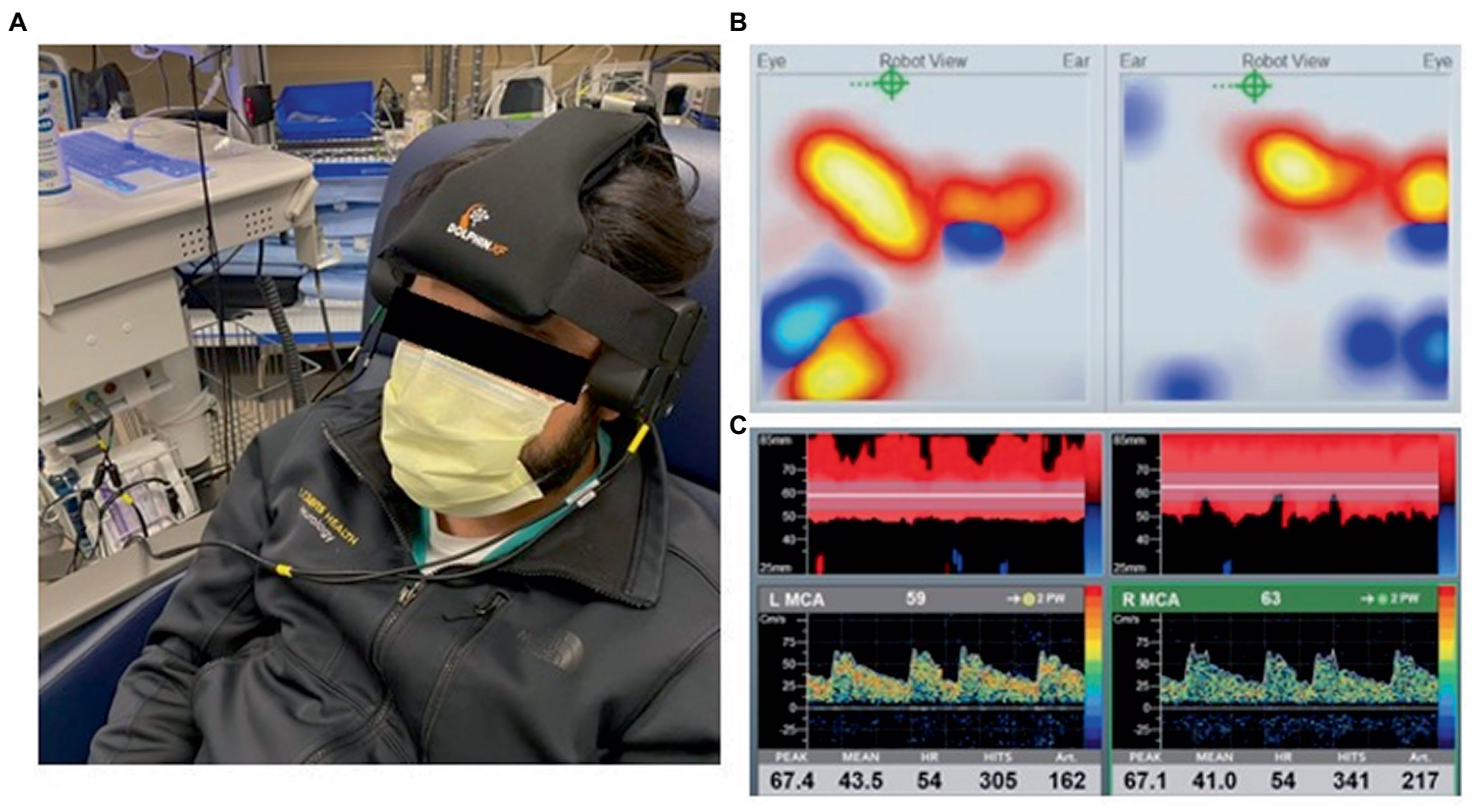
**(A)** Transcranial doppler (TCD) robotic headset placement. **(B)** Automated robotic scanning for intracranial vessel. **(C)** Continuous bilateral middle cerebral artery recordings with power M-mode and spectral doppler. Viasonix Dolphin/XF TCD Robotic Probe, Imaging Monitoring United States^®^.

While these point of care applications are very useful in clinical practice, TCD is often performed in an isolated or intermittent fashion which limits the ability to capture dynamic alterations in the FV waveform and longitudinal changes in cerebrovascular hemodynamics (94). Furthermore, TCD signal acquisition is highly operator dependent and technically challenging with ~10% of patients lacking adequate temporal acoustic windows (144). Despite these limitations, recent innovations in automated robotic technology have made prolonged monitoring with TCD more suitable for the ICU environment to derive flow based indices for risk stratification and goal directed therapies (Figure 6) (145).

### Pulsatility index, FV thresholds and prognosis

The degree of waveform resistance can be quantified by the Gosling pulsatility index (PI) [(PSV-EDV)/MFV] which has gained particular interest due to its association with ICP and inverse relationship with CPP in the setting of brain injury (146, 147). Though this correlation has been validated in several investigations, particularly in the extremes of ICP and CPP, PI is a complex parameter influenced by multiple different factors including distal cerebrovascular resistance (CVR), CPP, arterial blood pressure (ABP), pulse amplitude, heart rate and cerebral artery compliance, making direct parallels with ICP/CPP challenging (148–150). This shifting relationship is highlighted during a hyperventilation challenge where an expected increase in CPP occurs due to a drop in ICP however PI paradoxically increases as a result of arteriolar vasoconstriction and increased CVR (148). Notwithstanding these limitations, elevated PI above 1.3–1.5 in patients with TBI has been linked to increased risk of neurologic decline and worse outcome, particularly if measured within the first 24 h of admission, and predict the development of malignant cerebral edema, herniation and midline shift in patients with MCA territory ischemic stroke (151–153).

TCD derived CBFV measurements also provide useful information regarding the competency of cerebral perfusion, with low values suggestive of oligemia, and have been linearly correlated with CBF using computed tomography perfusion (CTP) in patients with diffuse traumatic injury patterns (154). A low-flow state defined as MCA MFV below 40 cm/s, occurs in over half of TBI patients during the first 24 h after injury, most often ipsilateral to focal pathology and correlates with the burden of cerebral hypoxia (PbtO_2_ <20 mmHg) (155, 156). The combination of elevated PI (suggesting increased resistance) with MFV 35–40 cm/s or EDV <20–25 cm/s (signifying impaired CBF) identifies TBI patients with particularly high risk of poor outcome and has even been validated in a mixed ICU population of patients with coma for mortality (152, 153, 157, 158). In addition to prognostication, TCD based thresholds may be used to recognize patients at risk for early deterioration and guide targeted therapy in hopes of preventing cerebral hypoperfusion. This has been exemplified in single center studies where mannitol administration in patients with hemispheric injury led to improvement in dangerously low MFV as well as aggressively treating abnormal TCD findings with hyperosmolar therapy, vasopressors and surgical intervention leading to normalization of TCD parameters in over 80% of patients (159, 160). Though external validation is required to better understand optimal treatment thresholds and the impact of TCD goal-directed therapy on outcomes, these studies illustrate how non-invasive TCD may provide insights into cerebral physiology at the bedside and inform personalized management decisions to limit secondary brain injury.

### Non-invasive ICP and CPP

The TCD waveform is responsive to changes in vascular tone and CBF with corresponding alterations in morphology in the setting of fluctuations in ICP and ABP (161, 162). Given this close relationship, there has been significant emphasis placed on developing non-invasive quantitative methods for ICP and CPP estimation (nICP and nCPP) based on TCD waveform morphological features (161). Initial efforts focused on PI as a quantitative measure of non-invasive ICP (nICP) given the strong correlation between the two parameters during periods of ICP elevation (148). The most favorable results were published by Bellner in 2014, expressed as nICP = 10.927 × PI – 1.284 with a 95% confidence interval of ±4.2 mmHg and strong correlation coefficient (*R* = 0.94), however these results could not be replicated in other patient populations (149, 163). Subsequent nICP investigations using PI have yielded less promising results with a wide range of confidence intervals, likely owing to factors other than ICP that impact PI, and suggest a limited role for PI in nICPP determination (161).

Several other quantitative approaches have focused on nCPP. This allows for nICP estimation using the mathematical relationship nICP = ABP – nCPP. These methodologies rely on TCD and ABP waveform analysis and include the diastolic flow velocity model (nICP_FVd), which leverages the close relationship between EDV and CPP, and the critical closing pressure (CrCP) model (nICP_CrCP) based on the concept of the minimum ABP required to prevent microvascular circulation and cessation of blood flow (161, 164, 165). A 2016 study from Cambridge United Kingdom compared these approaches in addition to PI derived nICP (nICP_PI) and a mathematical “black box (BB)” model (nICP_BB). Using a prospective TBI cohort with invasive ICP monitoring, researchers found that nICP_FVd, nICP_CrCP and nICP_BB generally performed well in ICP estimation with nICP_BB providing the most accurate ICP prediction with the least bias (166). In contrast, nICP_PI was the most sensitive to changes in ICP however proved to be the worst estimator of absolute ICP values.

Subsequent investigations using these non-invasive methods in different populations has produced mixed results with evidence of good correlation, though less accurate prediction, of absolute nICP and nCPP values (167, 168). The multicenter IMPRESSIT-2 study found nICP estimation based on nICP_FVd resulted in a high NPV for ICP >20 mmHg and >25 mmHg, 91.3 and 98.6%, respectively, though a concordance correlation between nICP and invasive ICP of only 33.3% (169). Conversely, in a recent study involving 100 TBI patients, nICP_FVd had essentially no agreement with invasive ICP (*R* = −0.17; 95% CI: −0.35, 0.03; *p* = 0.097) and a sensitivity of 0% for detecting ICP >20 mmHg, though notably few patients had elevations in ICP in this cohort (170). Similar findings have been reported in nCPP estimation in children with TBI using nICP_CrCP where the ability to identify CPP values below 70 mmHg was excellent (AUC = 0.91; 95% CI: 0.83–0.99) though overestimated true CPP by about 20 mmHg and was less precise for more clinically relevant thresholds of CPP < 60 mmHg and <50 mmHg (167). In another pediatric TBI study, both nICP_FVd and nICP_CrCP were compared against invasive monitoring and while there was a strong correlation between both models and CPP, each were also associated with wide limits of agreement and unable to discriminate CPP values <50–60 mmHg (171). Although the performance characteristics of current non-invasive TCD models preclude the ability to precisely measure ICP and CPP, they may still serve as a primary assessment tool in the acute stages of management or in patients who are not candidates for invasive monitoring. Furthermore, combining TCD parameters with other noninvasive non-invasive indices such as optic nerve sheath diameter and pupillometry may improve the diagnostic accuracy to identify patients with elevated ICP (172, 173).

### TCD derived autoregulatory indices

Given the close relationship of the TCD waveform to fluctuations in perfusion, evaluating for CA is made possible by way of a moving correlation coefficient to model the relationship between alterations in CBFV in response to slow wave changes in CPP. These indices including Mx, Sx, and Dx which correspond to TCD derived MFV, PSV, and EDV, respectively (174). In TBI patients, Mx is the most well-established index and appears to accurately capture dynamic CA fluctuations with evidence of a pressure passive state when significantly elevated and exhibits a moderate correlation to ICP-derived PRx (48, 162, 175). In particular, during elevations in ICP above 30 mmHg, there is an observed divergence which may be explained in part by the fundamental differences in how the two indexes relate to cerebrovascular physiology (48, 175, 176). In contrast, Sx appears to more closely approximate PRx, potentially related to increased systolic peaks and arterial pulsatility in the setting of elevated ICP, while Dx appears to possess limited clinical utility (45, 48). Both Mx and Sx correlate with mortality as well as neurologic outcome in TBI populations with evidence from several reports suggesting that Sx may possess superior outcome prediction characteristics (44, 48, 174). Using a small TBI cohort, Mx and Sx were found to have a parabolic relationship with CPP, thus allowing for determination of CPP_OPT_ with overall good agreement compared to PRx (42). Furthermore, by substituting ABP for CPP, Mx_a, and Sx_a can be determined with the potential to estimate PRx and CPP_OPT_ without the need for an ICP monitor and may provide a path forward for use of TCD-based advanced cerebral monitoring and goal directed therapy in a wider population of critically ill patients without invasive monitors (145, 174, 177).

Other potential advantages of TCD rely on the fact that it can interrogate different vascular beds and provide a better understanding of physiologic asymmetries, including CA capacity, compared to other global monitors. CBF is highly heterogeneous in patients following TBI with more frequent loss of CA ipsilateral to the site of injury and the overall magnitude of TCD-derived hemispheric CA asymmetry has been demonstrated to confer an increased risk of death and poor neurologic outcome (43, 178). In patients presenting with MCA stroke, the degree of CA dysregulation, as measured by the ipsilateral Mx_a, correlates with worse functional outcomes and larger overall final infarct volumes (179). Similar findings have been described in patients presenting with spontaneous intracerebral hemorrhage (ICH) where a gradual decline in CA capacity ipsilateral to the hemorrhage location over the first 5 days was associated with a decline in clinical exam and worse 90-day outcomes (180). In SAH, increases in Mx_a and Sx_a have been associated with the development of vasospasm in the ipsilateral hemisphere and combining radiographic vasospasm with increasing Mx_a from baseline over the first 7 days from ictus has been correlated with the development of DCI (181, 182).

Given advances in TCD technology, it is conceivable that CA could be monitored simultaneously in different hemispheres to allow for more precise evaluation of heterogeneous cerebral physiology and estimation of regional CPP_OPT_. Furthermore, unlike PRx, which is based on changes in ICP as a surrogate of CBV and relies on the intracranial pressure-volume relationship, TCD more directly measures CBF and can provide insights into dynamic CA changes even in the setting of highly compliant intracranial systems where PRx may not demonstrate significant fluctuations (35, 47). Despite these reasons for optimism, much work still needs to be done to validate TCD as a feasible continuous or semi-continuous monitoring technique as the majority of published studies report only snap-shots in time with no study reporting monitoring durations longer than 4 h (183). Furthermore, the vast majority of TCD derived CA reports are from data generated at the Addenbrooke’s Hospital in Cambridge United Kingdom, highlighting the need for external validation in different hospital settings and patient populations (183). Recently, a proposed protocol was published for a prospective multicenter study in severe TBI patients utilizing TCD with ICP/PbtO_2_ monitors with the goal of obtaining high-fidelity continuous data and correlate TCD based indices such as Mx/Mx_a, Sx/Sx_a, and CrCP with invasive parameters (94). This study and others like it may enhance our understanding of how TCD based indices may complement or alternatively serve as a surrogate for other MMM outputs and identify clinically relevant targets for future interventions.

## Near-infrared spectroscopy

Near-infrared spectroscopy (NIRS) is a non-invasive monitoring modality that can provide real-time information about regional cerebral oxygenation and CBF. The basis of this technology involves the emission of a near-infrared light source, in the range of 700–1,100 nm, to penetrate through the skin, cranium and most superficial few centimeters of brain (184). Depending on different tissue characteristics and cellular interfaces, the emitted light can either be scattered, reflected back to the detector, or absorbed by different chromophores such as protein, lipids and water (185). Hemoglobin is one such important organic macromolecule that exhibits differential absorption spectra characteristics depending on oxygenation status, thus allowing for determination of relative concentrations of oxyhemoglobin (HbO) and deoxyhemoglobin (HHb) in tissue according to the Modified Beer–Lambert law (186, 187). This technique provides estimates of total hemoglobin concentration (HbT) as well as the ratio of HbO to HbT called regional cerebral oxygen saturation (rSO_2_) or Tissue Oxygenation Index (TOI) depending on the manufacturer (185, 188). Similar to PbtO_2_, rSO_2_, and TOI reflect the balance between oxygen supply and demand within the distal arterial, venous and capillary territory and has demonstrated good correlation with invasively obtained absolute CBF values in critically ill patients (189–191). The majority of NIRS applications involve placing optodes over the forehead and measure signal over the frontal lobe gray matter and watershed zone of the anterior and middle cerebral arteries with the normal range of rSO_2_ being 55–80% (192, 193).

Low rSO_2_/TOI values, defined as <50–60% is concerning for ischemic insult in a single study demonstrating TOI thresholds of ≥75 and <55% correlating well with CPP values above and below 70 mmHg, respectively (192, 194). Though these thresholds serve as a reference, intra-patient optical measurement can vary significantly depending on cranial geometry and it is often more useful to monitor for relative changes in rSO_2_/TOI to detect cerebral ischemia (195). In TBI patients monitored with NIRS, cerebral hypoxia and hypoperfusion events are common even in the presence of accepted normal CPP range and has been correlated with more severe grade injury and higher likelihood of mortality (196, 197). NIRS has also been studied extensively in comparison to other modalities for cerebral oxygen assessment including to SjvO_2_, which is a global measure of cerebral oxygen supply and utilization, and exhibits a modest correlation with conflicting findings regarding sensitivity to changes in ABP, PaCO_2_, and CPP (191, 198, 199). Similarly when compared to PbtO_2_, NIRS has demonstrated variable levels of concordance with one study reporting an inability to detect clinically significant episodes of low PbtO_2_ with a high failure rate due to poor sensor-skin contact, scalp hematoma and subdural air after cranial procedures (185, 200). Conversely in a study with 42 TBI patients, NIRS and TCD demonstrated parallel findings with PbtO_2_, though more rapidly detected alterations in ABP and ICP, supporting the concept that no single bedside monitor represents the “gold standard” for cerebral oxygenation, with each device appearing to monitor different components of cerebral oxygenation, and may enhance diagnostic utility when used in complementary overlapping approaches (201, 202).

### Autoregulation indices

Multiple studies have explored the capacity of NIRS for non-invasive CA determination by correlating slow wave oscillations in NIRS derived parameters, such as rSO_2_, TOI, or HbT, with alterations in ABP or CPP resulting in the Cerebral oximetry index (COx), Tissue Oxygen Reactivity Index (TOx) and Total Hemoglobin Reactivity Index (THx), respectively (203, 204). These indices have been validated in piglet animal models, where they demonstrate a strong agreement with laser-doppler flow measurements and were capable of detecting both upper and lower limits of autoregulation during controlled titration of ABP (205–208). In clinical studies, NIRS based indices have been compared to ICP derived PRx with good overall correlation however a less robust agreement has been described in the presence of insufficient slow wave power or during periods of significant NIRS interhemispheric variance (203, 209).

TOx and COx are the most widely used parameters for CA estimation and demonstrate a strong correlation with TCD-derived Mx/Mxa (*r* = 0.55–0.81) in both operative and ICU based studies with thresholds of ~0.2–0.3 denoting loss of CA (210–213). Furthermore, combining Sx_a and TOx in SAH patients appears to strengthen the predictive capabilities for detection of DCI, likely due to measurement of different, though complementary, anatomic components of the cerebral vascular system for enhanced surveillance (214, 215). In mixed ICU populations, impaired CA as determined by TOx/COx has been correlated with the development of ICU delirium and all-cause mortality at 3 months presumably due to CBF dysregulation (216–218). Multiple investigations have also assessed the utility of NIRS in cardiac arrest patients, who often do not have invasive neuromonitoring and guidelines for hemodynamic management have traditionally focused on static thresholds (219). In these studies, higher TOx/COx values over the first 3 days is independently associated with an excess in mortality (220). Additionally several studies have demonstrated the feasibility of determining optimal MAP based on COx with evidence of a wide diversity of optimal thresholds across individuals, likely reflecting different cerebral injury patterns and impact of preexisting hypertension, with more favorable outcomes noted when the actual MAP more closely aligns with NIRS derived thresholds (219, 221, 222). These findings highlight the potential utility of NIRS technology to provide a foundation for non-invasive individualized hemodynamic management in patients with hypoxic ischemic brain injury. Similar findings have been reported in TBI patients using COx derived CPP_OPT_, which when compared to ORx, more strongly correlates with PRx derived thresholds with evidence that deviations between actual CPP and CPP_OPT_ of >10 mmHg are more likely to be associated with adverse outcomes (223).

Other studies have focused on THx, alternatively referred to as Hemoglobin Volume Index (HVx), which, in contrast to the CBF weighted TOx/COx, may be more reflective of CBV-based measurements in a similar fashion to PRx (193, 203). In a cohort of 40 TBI patients, THx demonstrated a significant association with PRx in individual recordings with ~50% of recordings suitable to determine optimal CPP and MAP with good agreement compared to PRx derived thresholds (204). In another TBI study, while correlation with PRx was similar between THx and TOx, albeit with large limits of agreement, THx demonstrated poor correlation with Mx supporting the notion that NIRS may provide information about distinctive features of CBF compared to TCD based parameters with the potential for complementary assessment in clinical situations where invasive monitoring is not otherwise indicated or safe (203).

Similar to TCD, NIRS has the advantage of being completely non-invasive, portable and capable of providing regional assessment of cerebral physiology and oxygenation. In contrast to TCD however, NIRS has the added benefit of being non-operator dependent, easily applied in the operative and ICU environments, without the need for frequent calibration and capable of generating continuous monitoring data (185, 187). Multiple different NIRS techniques have been developed with particular strengths and limitations in addition to proprietary algorithms and indices. For example, depending on the manufacturer, the ratio of HbO relative to HbT can be described by rSO_2_ as well as TOI, ScO_2_, and SpO_2_ with similar, though not completely interchangeable, values (188). The most common techniques include fixed wavelength, spatially-resolved, frequency-resolved, time-resolved and diffuse correlation spectroscopy techniques which are beyond the scope of this review to individually detail though differ in their associated cost, depth of measurement, precision and ability to provide complementary information about absolute CBF as well as CMRO_2_ (187).

Though advances in NIRS technology provides grounds for optimism regarding the development of non-invasive assessment of cerebral hemodynamics, oxygenation and metabolism, there are several limitations that must be addressed. First, NIRS requires a close spatial relationship between the cortex and cranium and is prone to inaccurate readings in the setting of post-operative pneumocephalus, skin pigmentation, scalp edema, extracranial hemodynamics, frontal contusions, and hemorrhage which are all common in neuro-ICU populations (224–226). As a regional monitor, it is only sensitive for changes in the superficial cortical structures, often restricted to the frontal brain region, and is unable to detect distant ischemic events (204). As a complex physiologic parameter, NIRS-based cerebral oxygenation is also influenced by a multitude of physiologic factors thus limiting attempts to draw direct conclusions about cerebral respiration and CBF. Efforts to establish clear prognostic thresholds are hindered by a multitude of different proprietary indices and conflicting reports across diverse pathologies and patient populations (185, 195, 211, 227). While the assortment of different NIRS based technologies allows for focused investigation and novel insights into aspects of cerebral physiology, the reporting of multiple similar yet distinct parameters limits the generalizability of scientific findings across different devices (228). The role of NIRS monitoring in neurocritical care populations has yet to be verified in large, multicenter trials, and at this time should continue to be explored under the context of clinical research. Further investigation is required to determine optimal NIRS based technology, monitoring parameters, and thresholds to optimize CA and potentially improve neurologic outcomes across a spectrum of ABI patients with particular focus in populations where invasive monitoring is not routinely performed or is otherwise contraindicated.

## Current limitations and future directions

While it is evident that MMM allows for improved characterization of complex and often interrelated neurophysiologic determinants, considerable heterogeneity exists in the application and utilization of advanced neuromonitoring due to lack of familiarity, cost of high-resolution data acquisition and uncertainty regarding clinical efficacy (4, 9). Though there is accumulating data to suggest that targeted management of cerebral parameters such as pressure, blood flow and metabolism may prevent neurologic deterioration, there is a lack of high-quality prospective data to support widespread implementation of protocolized monitoring strategies. Even when considering CA and the advances in continuous assessment capabilities, current therapeutic interventions appear entirely inadequate to correct dysregulated CA thus underscoring the gap in our understanding of molecular and cellular pathways involved in cerebral homeostasis (229–231). Future research will need to focus on improved methods of data collection with more clearly defined pathologies, clinically meaningful outcomes, and assessment of secondary neurologic injury. As the number of MMM strategies expands, integrative approaches with a focus on standardized methods of real-time data visualization and analysis will be needed to translate these approaches effectively from research into clinical practice (232). Additionally, leveraging machine learning technology to provide automated detection of multidimensional physiologic patterns may help forecast deleterious events and provide an opportunity to intervene before there is risk of permanent injury thereby engaging in preventive rather than reactionary treatment approaches (233–235).

## Conclusion

There is ample mounting evidence that invasive and non-invasive neuromonitoring techniques provide instrumental information regarding the cerebral physiome that otherwise would not be possible with standard bedside clinical assessment and imaging alone. Integration of various neuromonitoring modalities using a central interface at the bedside allows clinicians to monitor physiologic data in real time, which, not only facilitates improved understanding of complex neurophysiologic processes but can also guide targeted interventions to restore cerebral homeostasis. Goal directed therapy using neuromonitoring tools can however, only be realized when multimodal data is collected and integrated using high-resolution time-synchronized data collection and archiving system. Such technological advances have the potential to transition neurocritical care from standardized “one size fits all” treatment paradigm based on epidemiologic studies, to a focus on precision medicine centered on continuously adjusted individualized thresholds with the goal of improving patient outcomes. Substantial need still exists for innovative methods of enhanced data integration and interpretation as well as high-quality outcome-based studies before widespread application and acceptance can be expected.

## Author contributions

JV designed and conceptualized the manuscript, literature review, interpretation and summarization of data, drafting of the manuscript, and final approval of the manuscript. NL substantial contribution in the design and draft of the initial manuscript, literature review, interpretation and summarization of data, and approval of the final manuscript. SM designed and conceptualized the manuscript, literature review, interpretation and summarization of data, critical revision of the manuscript for important intellectual content, and final approval of the manuscript. All authors contributed to the article and approved the submitted version.

## Conflict of interest

The authors declare that the research was conducted in the absence of any commercial or financial relationships that could be construed as a potential conflict of interest.

## Publisher’s note

All claims expressed in this article are solely those of the authors and do not necessarily represent those of their affiliated organizations, or those of the publisher, the editors and the reviewers. Any product that may be evaluated in this article, or claim that may be made by its manufacturer, is not guaranteed or endorsed by the publisher.
